# Fixed‐jaw technique to improve IMRT plan quality for the treatment of cervical and upper thoracic esophageal cancer

**DOI:** 10.1002/acm2.12704

**Published:** 2019-08-28

**Authors:** Wei Song, Hong Lu, Jie Liu, Di Zhao, Jun Ma, Biyun Zhang, Dahai Yu, Xinchen Sun, Jinkai Li

**Affiliations:** ^1^ Department of Radiation Oncology Affiliated Hospital of Nanjing University of Chinese Medicine Nanjing China; ^2^ Department of Radiation Oncology the First Affiliated Hospital of Nanjing Medical University Nanjing China

**Keywords:** cervical and upper thoracic esophageal cancer, dose fall‐off, dosimetry, fixed‐jaw technique, intensity‐modulated radiotherapy

## Abstract

The purpose of this study was to investigate the potential advantages of the fixed‐jaw technique (FJT) over the conventional split‐field technique (SFT) for cervical and upper thoracic esophageal cancer (EC) patients treated with intensity‐modulated radiotherapy. The SFT and FJT plans were generated for 15 patients with cervical and upper thoracic EC. Dosimetric parameters and delivery efficiency were compared. An area ratio (AR) of the jaw opening to multileaf collimator (MLC) aperture weighted by the number of monitor units (MUs) was defined to evaluate the impact of the transmission through the MLC on the dose gradient outside the PTV50.4, and the correlation between the gradient index (GI) and AR was analyzed. The FJT plans achieved a better GI and AR (*P* < 0.001). There was a positive correlation between the GI and AR in the FJT (*r* = 0.883, *P* < 0.001) and SFT plans (*r* = 0.836, *P* < 0.001), respectively. Moreover, the mean dose (D_mean_), V_5Gy_–V_40Gy_ for the lungs and the D_mean_, V_5Gy_–V_50Gy_ for the body‐PTV50.4 in the FJT plans were lower than those in the SFT plans (*P* < 0.05). The FJT plans demonstrated a reduction trend in the doses to the spinal cord PRV and heart, but only the difference in the heart D_mean_ reached statistical significance (*P* < 0.05). The FJT plans reduced the number of MUs and subfields by 5.5% and 17.9% and slightly shortened the delivery time by 0.23 min (*P* < 0.05). The gamma‐index passing rates were above 95% for both plans. The FJT combined with target splitting can provide superior organs at risk sparing and similar target coverage without compromising delivery efficiency and should be a preferred intensity‐modulated radiotherapy planning method for cervical and upper thoracic EC patients.

## 1 INTRODUCTION

Esophageal cancer (EC) is the eighth most common cancer and the sixth leading cause of cancer‐related death worldwide, with over 450 000 new cases and 400 000 deaths per year.^1^ Since the majority of patients are diagnosed with locally advanced disease and are ineligible for surgery, radiotherapy plays an important role in the definitive management of EC,^2^ especially for disease located in the cervical and upper thoracic regions. However, radiotherapy planning for cervical and upper thoracic EC is technically challenging owing to the dose‐limiting adjacent critical structures and rapid changes in body contour and tumor depth.^3^, ^4^ Compounding the issue is the large and complex target volume when electively treating the high‐risk lymph nodal regions.

Intensity‐modulated radiotherapy (IMRT) is an advanced radiation delivery technique that can produce highly conformal dose distribution to the tumor while minimizing irradiation of surrounding healthy tissues. Multiple studies have demonstrated its dosimetric and clinical advantages over three‐dimensional conformal radiotherapy (3D‐CRT) for EC patients.^4^, ^5^ However, IMRT is generally associated with a larger volume of low‐dose spread to normal tissues, in part due to the transmitted radiation through the multileaf collimator (MLC).^6^, ^7^ Clinical studies have found that the volumes of the lung receiving low doses were strongly associated with the incidence of radiation pneumonitis (RP),^8^, ^9^ which is a major concern for EC patients receiving radiotherapy. It may not only cause serious pulmonary injury but also limit dose escalation strategies.^10^, ^11^ Therefore, great consideration should be given to minimizing the low‐dose exposure of the lung during IMRT planning. Recently, a fixed‐jaw technique (FJT) has been proposed to reduce MLC transmission in IMRT by properly adjusting and fixing the secondary collimator jaw positions during plan optimization, which has been demonstrated to be effective in limiting low doses spreading to normal tissues while maintaining the target coverage for gynecological cancer,^12^ breast cancer,^13^ lung cancer,^14^ and head and neck cancer.^15^ However, to the best of our knowledge, the application of FJT to the treatment of cervical and upper thoracic EC has not yet been reported in the literature. Considering the complex target and adjacent critical structure geometries for EC located in the cervical and upper thoracic regions, the impact of FJT on IMRT plan quality for these patients remains unclear.

The purpose of this study was to investigate the possibility of using FJT combined with target splitting to improve the IMRT plan quality for cervical and upper thoracic EC. The potential advantages of this technique over the conventional split‐field technique (SFT) were evaluated by comparing the target coverage, protection for organs at risk (OARs), and delivery efficiency.

## 2 MATERIALS AND METHODS

### 2.1 Patient characteristics

Fifteen medically inoperable patients with cervical and upper thoracic EC previously treated with definitive IMRT at our department were included in this study. The patients consisted of 13 men and 2 women. Their median age was 63 yr old (range: 46 to 76 yr). All patients were histologically or cytologically confirmed to have squamous cell carcinoma. According to the clinical staging of esophageal carcinoma receiving nonsurgical treatment,^16^ three patients had stage Ⅰ disease, eight had stage Ⅱ disease, and four had stage Ⅲ disease. Among the 15 patients, there were six cases with the primary tumor located in the cervical esophagus and nine in the upper thoracic esophagus.

### 2.2 CT simulation, target, and OAR delineation

All patients were immobilized with a head, neck, and shoulders thermoplastic mask in a supine position with both arms along the body. The CT simulation was performed with a 5‐mm slice thickness from the cranial base to the lower edge of the liver using a Philips Brilliance Big Bore CT scanner (Philips Medical Systems, Inc., Cleveland, OH, USA). The CT images were then transferred to the Eclipse treatment planning system (TPS) (versions 8.6.23, Varian Medical Systems, Palo Alto, CA, USA) for treatment planning delineation. The gross tumor volume (GTV), clinical target volume (CTV), and planning target volume (PTV) were contoured by a senior physician. The GTV was defined as any visible primary tumor and metastatic lymph nodes by the CT, barium esophagogram, and endoscopic examination. The CTV60 was defined as the GTV with 3‐cm margins of proximal and distal normal esophagus and a 0.5‐cm radial margin. The CTV50.4 comprised the CTV60 and elective regional lymph nodes in the bilateral supraclavicular and superior mediastinal region. The PTV50.4 (median volume: 376.63 cm^3^; range: 237.32 to 668.95 cm^3^) and PTV60 (median volume: 102.62 cm^3^; range: 68.31 to 228.10 cm^3^) were generated by adding a 0.5‐cm isotropic margin to the CTV50.4 and CTV60, respectively. The lungs, spinal cord planning organ at risk volume (spinal cord PRV, spinal cord with a 5‐mm margin), heart, and body volume minus PTV50.4 (body‐PTV50.4) were contoured as OARs.

### 2.3 Treatment planning

Treatment planning was performed on the Eclipse TPS using the configured 6‐MV photon beam data for a Varian Clinac iX linear accelerator. The accelerator is equipped with a Millennium 120 MLC, which has 40 central leaf pairs with a projected width of 5 mm and 20 outer leaf pairs with a projected width of 10 mm at the isocenter. Two static gantry IMRT plans, that is, the SFT plan and the FJT plan, were generated for each patient using a simultaneously integrated boost scheme. The prescribed dose was 50.4 Gy in 28 fractions to the PTV50.4 and 60 Gy in 28 fractions to the PTV60. Both plans employed an eight‐field beam arrangement (285°, 300°, 330°, 0°, 30°, 60°, 145°, and 180° in the IEC61217 scale), which was chosen to maximally spare the lung while ensuring the target dose coverage.

**Figure 1:**
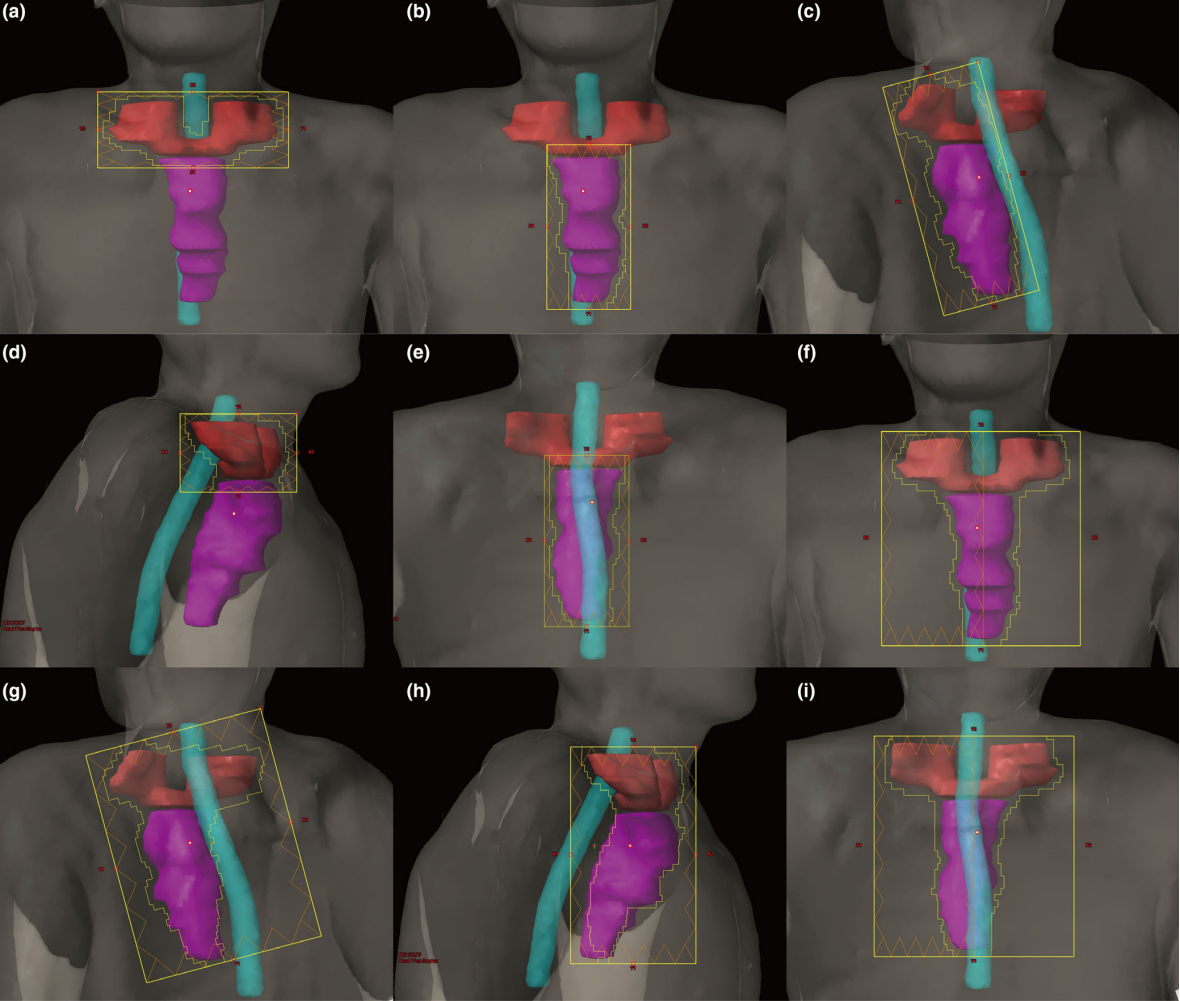
The beam's‐eye view at the gantry angle of 0° with a collimator angle of 90° (a), at the gantry angle of 0° with a collimator angle of 0° (b), at the gantry angle of 145° (c), 285° (d), 180° (e) in the fixed‐jaw technique plan and at the gantry angle of 0° (f), 145° (g), 285° (h), 180° (i) in the split‐field technique plan for patient number 9, showing the jaw opening (yellow rectangle), multileaf collimator aperture (irregular yellow outline), field‐splitting location (brown vertical line), PTV50.4‐sup (red), PTV50.4‐inf (purple), and spinal cord PRV (cyan).

In the SFT plan, the Eclipse TPS automatically determined the collimator jaw positions to cover the entire PTV50.4 in each beam direction. Treatment fields wider than 14.5 cm were split into two or more subfields by the TPS because the maximum travel distance of MLC leaves in one carriage group were limited to 14.5 cm. To minimize the transmitted radiation to the surrounding OARs in the FJT plan, we split the PTV50.4 into a superior part (PTV50.4‐sup) and an inferior part (PTV50.4‐inf) at the level of the sternal notch, and the collimator jaw positions were manually adjusted and fixed to modify the jaw opening of each field for these two parts. Figure 1 illustrates the jaw positions in the FJT and SFT plan for a representative patient. Several strategies were employed to determine the jaw positions as follows: (a) a minimal margin of 7 mm from the PTV50.4 to each jaw edge was kept to ensure the dose coverage at the edge of PTV50.4;^17^ (b) to minimize the volume of normal tissues surrounding the PTV50.4 shielded by MLC alone at the gantry angle of 0°, the large field was manually split into two subfields with a collimator rotation of 90° to maintain the number of subfields. One of the subfields covered the PTV50.4‐sup [Fig. 1(a)], and the other one covered the PTV50.4‐inf [Fig. 1(b)]. The two jaw openings were allowed to overlap 2 cm in the superior and inferior direction to feather their junction; (c) for other beam directions such as the gantry angle of 145°, 180°, and 285°, some parts of the PTV50.4 further away from the radiation source were manually shielded with the collimator jaws to further reduce the volume of surrounding normal tissues exposed to the transmission through the MLC. For example, the contralateral supraclavicular region was shielded with one collimator jaw at the gantry angle of 145° [Fig. 1(c)]. The field at the gantry angle 285° only covered the PTV50.4‐sup [Fig. 1(d)], whereas the field at the gantry angle of 180° only covered the PTV50.4‐inf [Fig. 1(e)]. Similarly, the jaw positions for these two fields were extended 1 cm across the junction plane for the purpose of dose feathering. Figures 1(f)–1(i) shows the jaw positions for the corresponding fields in the SFT plan for the same patient.

The optimization parameters were kept the same for both plans. The planning goal for the PTV50.4 and PTV60 was 95% of the target volume should be covered by 100% of the prescribed dose. The dose constraints for the OARs were as follows: lung, the mean dose (D_mean_) < 13 Gy, V_5Gy_ ≤ 55% (i.e., the percentage volume of the lung receiving ≥5 Gy), V_20Gy_ ≤ 28%, and V_30Gy_ ≤ 20%; spinal cord PRV, D_1%_ (the minimal dose received by the hottest 1% volume) ≤ 45 Gy; heart, V_30Gy_ ≤ 40%. Two ring structures were generated to conform the prescription dose to the target. The first ring was defined as a 4‐cm‐width ring structure around the PTV50.4. The second ring was defined as the body volume outside the first ring. The sliding window technique was selected for treatment delivery at a dose rate of 400 MU/min. The dose distribution was calculated using the analytical anisotropic algorithm with a 2.5‐mm grid spacing including tissue heterogeneity corrections. Each plan was normalized to cover 95% of the PTV50.4 with 100% of the prescribed dose. To maintain the consistency of the plan quality, all plans were designed by a dosimetrist with 8 yr of clinical experience in IMRT planning.

### 2.4 Plan evaluation

Based on the dose‐volume histogram (DVH) data automatically extracted from the DICOM plan files with an in‐house developed MATLAB (The MathWorks, Natick, MA, USA) program, the D_2%_, D_98%_, D_50%_, conformity index (CI), homogeneity index (HI) for the PTV50.4 and PTV60, V_100%_ for the PTV60, and gradient index (GI) for the PTV50.4 were calculated and compared between the two plans.

The CI was calculated as follows:^18^

(1) $$\text{CI}=\frac{\text{TV}\times \text{PIV}}{{\text{TV}}_{\text{PIV}}^{2}}$$

where TV is the target volume, PIV is the prescription isodose volume, and TV_PIV_ is the target volume enclosed by PIV. A CI closer to 1 indicates a better target conformity.

The HI was calculated as follows:^18^

(2) $$\text{HI}=\frac{D_{2\%}-D_{98\%}}{D_{50\%}}$$

An HI close to zero indicates an ideal target dose homogeneity.

The GI was defined as follows:

(3) $$\text{GI}=R_{50\%}-R_{100\%}$$

where *R*_100%_ is the equivalent sphere radius of the prescription dose volume, and *R*_50%_ is the equivalent sphere radius of the half prescription dose volume.^19^ A smaller GI indicates a sharper dose fall‐off outside the target volume.

To compare the volume of surrounding normal tissues shielded by the MLC alone in both plans, an area ratio (AR) of the jaw opening to MLC aperture weighted by the number of MUs was defined as follows:

(4) $$\text{AR}=\frac{\sum_{i=1}^{n}{\text{Sjaw}}_{i}\times {\text{MU}}_{i}}{\sum_{i=1}^{n}{\text{SMLC}}_{i}\times {\text{MU}}_{i}}$$

where Sjaw_i_ and SMLC_i_ represent the area of the jaw opening and the MLC aperture at the i‐th gantry angle, respectively. MU_i_ is the number of MUs at the i‐th gantry angle. n is the total number of subfields in each plan. An AR close to 1 indicates almost no volume of normal tissues surrounding the PTV50.4 receiving the transmission through the MLC. The MLC aperture was determined by the most retracted positions of each leaf across all segments,^20^ which is approximately equal to the projected area of the PTV50.4 inside the jaw opening at the isocenter. The aforementioned MATLAB program was employed to calculate the area of the MLC aperture based on the MLC sequence information extracted from the DICOM plan files.

To evaluate OAR sparing, DVH parameters for the lung (D_mean_, V_5Gy_, V_10Gy_, V_13Gy_, V_20Gy_, V_30Gy_, and V_40Gy_), spinal cord PRV (D_1%_), heart (D_mean_, V_30Gy_, and V_40Gy_), body‐PTV50.4 (D_mean_, V_5Gy_, V_10Gy_, V_20Gy_, V_30Gy_, V_40Gy_, and V_50Gy_) were calculated and compared.

In addition, both plans were delivered to a phantom in clinical mode to evaluate the treatment delivery efficiency. The number of MUs, number of subfields, and delivery time were recorded according to the ARIA 10 record and verify system (Varian Medical Systems, Palo Alto, CA, USA).

### 2.5 Plan verification

Plan verification for the FJT and SFT plans was performed with the two‐dimensional (2D)‐Array Seven29 and RW3 slab phantom (PTW, Freiburg, Germany). The 2D‐ARRAY Seven29 was set up under 5 cm of solid water with an 8‐cm solid water backscatter. The verification plans were created by separately transferring each treatment field of the patient plan to the verification phantom using the Eclipse TPS. All treatment parameters in the verification plan were the same as the original patient plan, except that the gantry angle was set to 0° for all fields. The measured dose distribution within the effective measuring plane of the 2D‐Array was compared with the TPS calculation using the gamma‐index method with a criterion of 3%/3 mm and a threshold dose of 10% of the maximum dose.

### 2.6 Statistical analysis

The statistical analysis was performed using SPSS Statistics19.0 software (IBM Corp., Armonk, NY, USA). The Shapiro–Wilk test showed that all the parameters were normally distributed. Differences between the two plans were analyzed using the paired *t*‐test. All the parameters were reported as the mean ± standard deviation (SD). The correlation between the GI and AR was evaluated using the Pearson correlation test. A *P* <0.05 (two‐tailed) was considered statistically significant.

## 3 RESULTS

**Figure 2:**
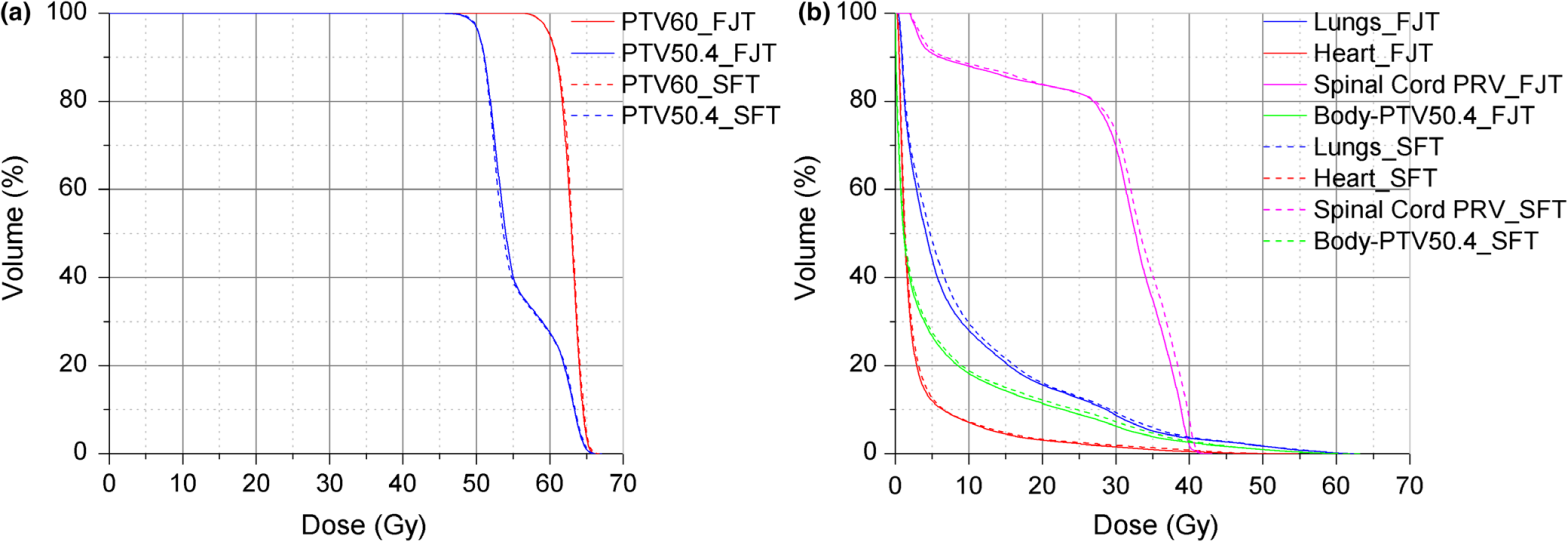
Dose‐volume histogram comparison for the planning target volume (a) and organs at risks (b) between the two techniques for patient number 9.

**Figure 3:**
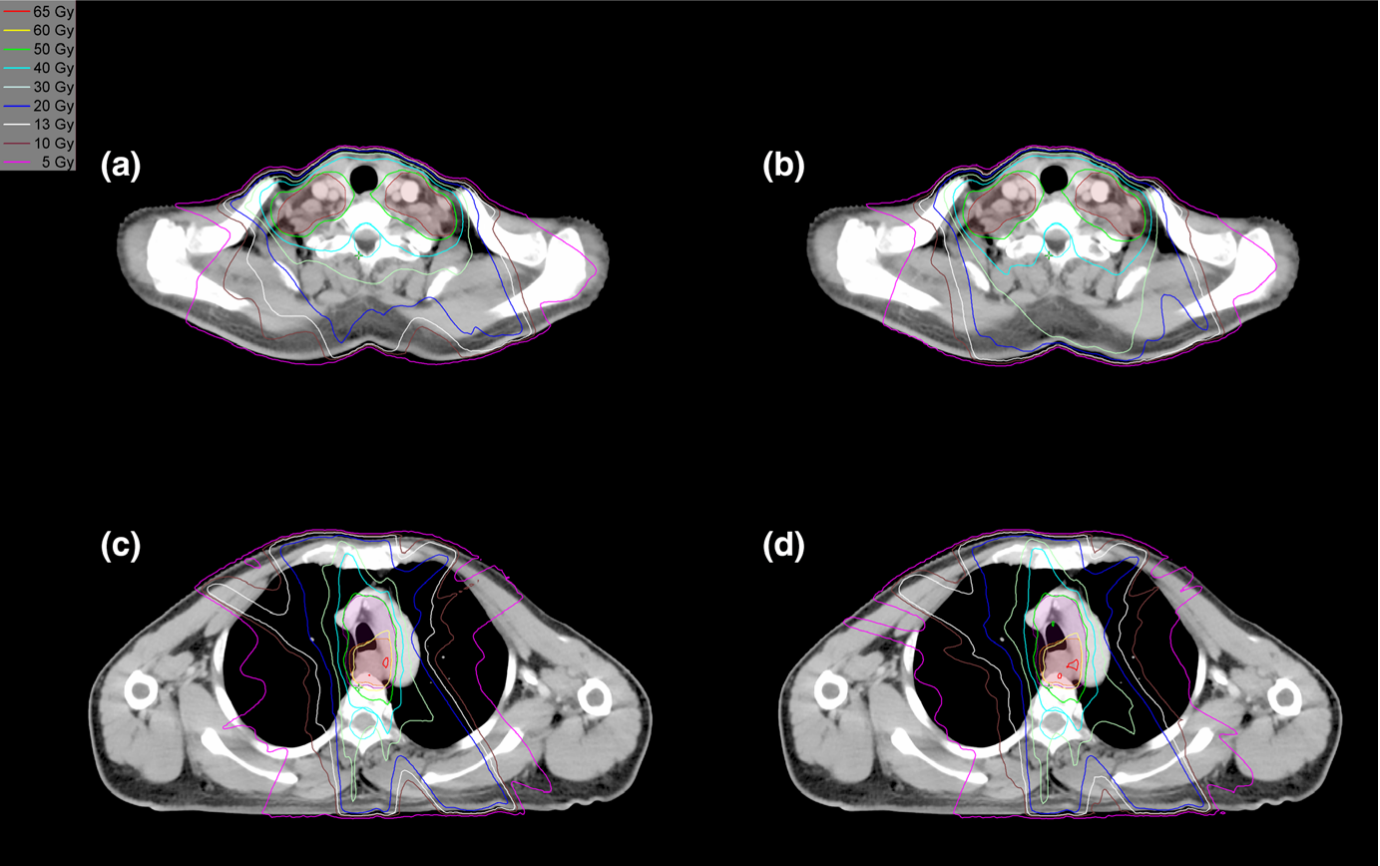
Comparison of the dose distributions in the supraclavicular region (a, b) and upper thoracic region (c, d) of the fixed‐jaw technique plan (a, c) and split‐field technique plan (b, d) for patient number 9. Orange: PTV60; red: PTV50.4‐sup; purple: PTV50.4‐inf.

Both the FJT and SFT plans were clinically acceptable. Figure 2 shows the DVH comparison of the two plans for a representative patient. The FJT plan reduced the low‐ and intermediate‐dose exposure of OARs, while providing comparable target dose coverage. Figure 3 displays the dose distributions of the same patient. The volumes of the body receiving different dose levels from 5 to 40 Gy were markedly reduced in the FJT plan compared to the SFT plan.

Table 1 summarizes the comparison of the PTV dosimetric parameters of the FJT and SFT plans for the 15 patients. The D_2%_, D_98%_, D_50%,_ CI, HI, and V_100%_ for the PTV60 and PTV50.4 were similar between the two techniques, with no significant difference (*P*> 0.05).

**Table 1:** Comparison of the planning target volume (PTV) dosimetric parameters of the two techniques (mean ± SD).

Variable	FJT	SFT	Difference	*P* value
PTV60				
D_2%_ (Gy)	64.75 ± 0.74	64.90 ± 0.78	−0.15	0.398
D_98%_ (Gy)	59.03 ± 0.21	59.04 ± 0.22	−0.01	0.821
D_50%_ (Gy)	62.47 ± 0.44	62.59 ± 0.46	−0.12	0.273
CI	1.28 ± 0.05	1.28 ± 0.05	0.00	0.667
HI	0.09 ± 0.01	0.09 ± 0.01	0.00	0.413
V_100%_ (%)	95.37 ± 0.67	95.64 ± 0.79	−0.27	0.501
PTV50.4				
D_2%_ (Gy)	63.95 ± 0.62	64.11 ± 0.67	−0.16	0.284
D_98%_ (Gy)	49.56 ± 0.19	49.58 ± 0.19	−0.02	0.703
D_50%_ (Gy)	55.71 ± 0.38	55.80 ± 0.44	−0.09	0.136
CI	1.37 ± 0.06	1.37 ± 0.06	0.00	0.555
HI	0.26 ± 0.02	0.26 ± 0.02	0.00	0.331

CI, conformity index; FJT, fixed‐jaw technique; HI, homogeneity index; SFT, split‐field technique.

**Figure 4:**
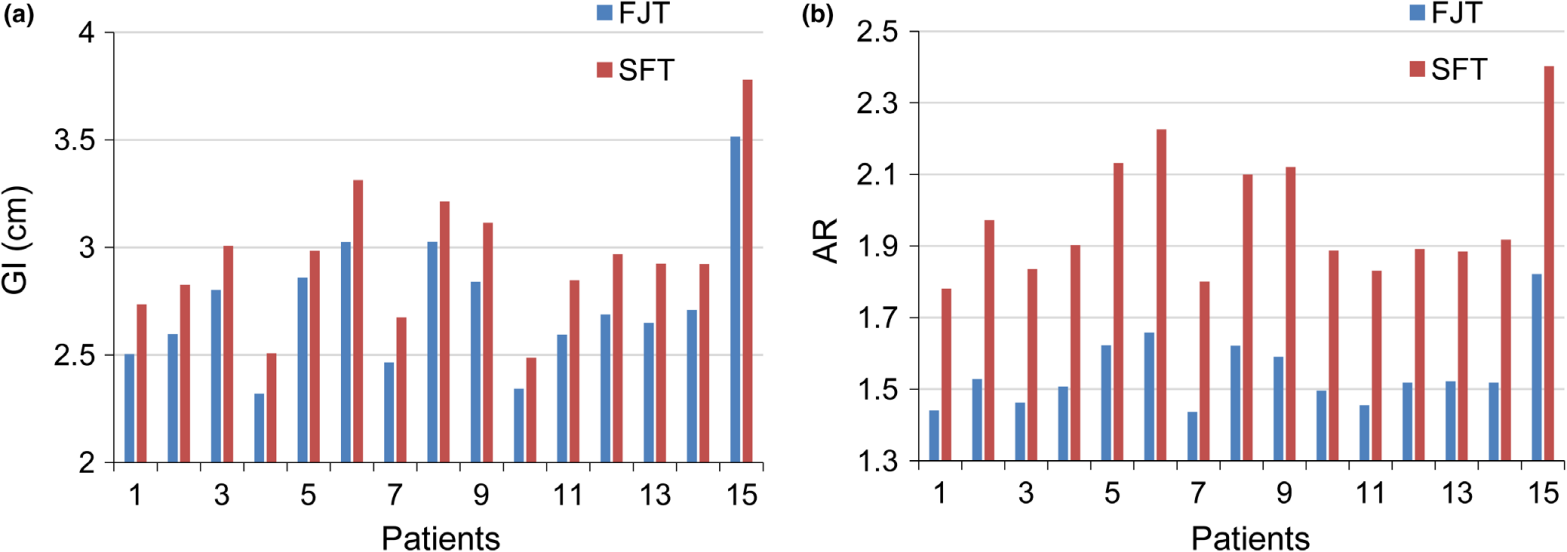
Comparison of the gradient index (a) and area ratio (b) of the two techniques for each patient.

**Figure 5:**
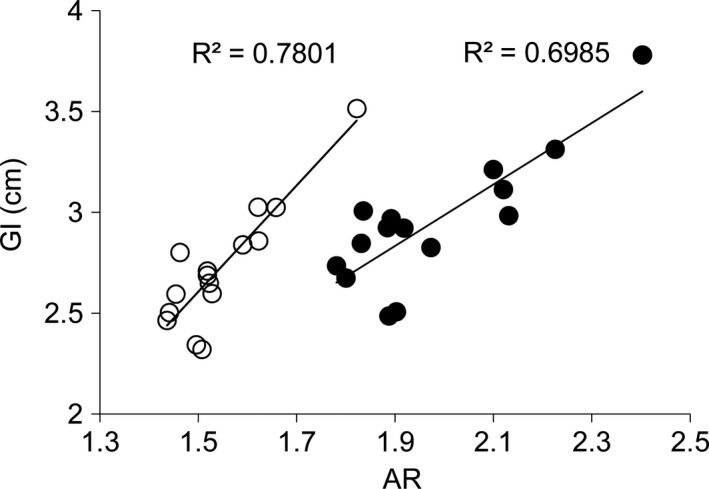
Correlation between the gradient index and area ratio in the fixed‐jaw technique plans (open circles) and split‐field technique plans (filled circles), respectively.

The FJT plans achieved a better GI and AR (2.73 ± 0.31 cm and 1.55 ± 0.10) than the SFT plans (2.95 ± 0.33 cm and 1.98 ± 0.18) (*P* < 0.001). As shown in Fig. 4, the GI and AR of the FJT plan were evidently improved for each patient. Interestingly, Fig. 5 shows a positive correlation between the GI and AR in the FJT plans (*r* = 0.883, *P* < 0.001) and the SFT plans (*r* = 0.836, *P* < 0.001), respectively, indicating that reducing the volume of surrounding normal tissues receiving the transmission through the MLC contributed to the steeper dose fall‐off outside the PTV50.4.

Table 2 summarizes the comparison of the OAR dosimetric parameters of the two techniques for all patients. The FJT plans demonstrated great advantages in reducing the volume of the lung and body‐PTV50.4 receiving different dose levels. The D_mean_, V_5Gy_, V_10Gy_, V_13Gy_, V_20Gy_,V_30Gy_, and V_40Gy_ for the lung, and the D_mean_, V_5Gy_, V_10Gy_, V_20Gy_, V_30Gy_, V_40Gy_, and V_50Gy_ for the body‐PTV50.4 in the FJT plans were significantly lower than those in the SFT plans (*P* < 0.05). Regarding the other OARs, the D_1%_ to the spinal cord PRV, and the D_mean_, V_30Gy_, and V_40Gy_ of the heart in the FJT plans were lower than those in the SFT plans, but only the difference in the heart D_mean_ reached statistical significance (*P* < 0.05). Since most heart volumes were located outside of the treatment field, the doses to the heart were considerably lower in both plans and should be less of a concern. The dose to the spinal cord PRV was also within the tolerance level.

**Table 2:** Comparison of the organs at risk dosimetric parameters of the two techniques.

Variable	FJT	SFT	Difference	*P* value
Spinal cord PRV				
D_1%_ (Gy)	41.22 ± 0.46	41.28 ± 0.62	−0.06	0.526
Lung				
D_mean_ (Gy)	8.26 ± 1.24	8.89 ± 1.40	−0.63	0.000
V_5Gy_ (%)	40.03 ± 5.69	43.80 ± 7.29	−3.77	0.000
V_10Gy_ (%)	26.14 ± 3.60	28.27 ± 3.96	−2.13	0.000
V_13Gy_ (%)	20.94 ± 2.86	22.52 ± 3.31	−1.58	0.000
V_20Gy_ (%)	13.51 ± 2.18	14.39 ± 2.42	−0.88	0.000
V_30Gy_ (%)	6.62 ± 1.71	7.38 ± 2.01	−0.76	0.001
V_40Gy_ (%)	3.04 ± 1.45	3.62 ± 1.69	−0.58	0.014
Heart				
D_mean_ (Gy)	1.88 ± 1.96	2.02 ± 2.08	−0.14	0.007
V_30Gy_ (%)	0.89 ± 2.48	0.97 ± 2.64	−0.08	0.174
V_40Gy_ (%)	0.40 ± 1.38	0.45 ± 1.44	−0.05	0.179
Body‐PTV50.4				
D_mean_ (Gy)	5.28 ± 0.72	5.66 ± 0.78	−0.38	0.000
V_5Gy_ (%)	24.19 ± 1.79	25.13 ± 2.10	−0.94	0.000
V_10Gy_ (%)	16.95 ± 1.32	17.46 ± 1.50	−0.51	0.000
V_20Gy_ (%)	10.57 ± 1.01	11.20 ± 1.02	−0.63	0.000
V_30Gy_ (%)	5.67 ± 0.87	6.52 ± 0.97	−0.85	0.000
V_40Gy_ (%)	2.58 ± 0.54	3.26 ± 0.62	−0.68	0.000
V_50Gy_ (%)	1.03 ± 0.35	1.18 ± 0.41	−0.15	0.000

FJT, fixed‐jaw technique; PTV, planning target volume; SFT, split‐field technique.

As shown in Table 3, the number of MUs and subfields for the FJT plans (1060.44 ± 114.47 and 9.71 ± 1.16) were significantly reduced by 5.5% and 17.9%, respectively, compared to those for the SFT plans (1122.19 ± 149.70 and 11.83 ± 1.51) (*P* < 0.05). However, the FJT plans only slightly shortened the delivery time by 0.23 min (*P* < 0.05), owing to the increased time of collimator rotation at the gantry angle of 0°. The plan verification results showed that the gamma‐index passing rates for both plans (99.27 ± 1.25% vs. 98.99 ± 1.11%) were above 95%, with no significant difference (*P* = 0.449).

**Table 3:** Comparison of the delivery efficiency of the two techniques.

Variable	FJT	SFT	Difference	*P* value
MUs	1060.44 ± 114.47	1122.19 ± 149.70	−61.75	0.006
Subfields	9.71 ± 1.16	11.83 ± 1.51	−2.12	0.000
Delivery time (min)	5.52 ± 0.27	5.75 ± 0.41	−0.23	0.027

FJT, fixed‐jaw technique; SFT, split‐field technique.

## 4 DISCUSSION

Owing to the complexity of the target and surrounding anatomy, radiotherapy planning for cervical and upper thoracic EC is very challenging.^4^ IMRT has a much greater potential for dose sculpting and normal tissue sparing than 3D‐CRT and has been widely adopted for EC treatment in clinical practice.^2^, ^5^ However, IMRT generally produces widely distributed low‐dose levels in the normal tissues surrounding the PTV, especially for the treatment of large and complex targets. In this study, we applied the FJT combined with target splitting to the treatment of cervical and upper thoracic EC in order to minimize the low‐dose exposure of the normal tissues while preserving the target coverage and delivery efficiency.

Our data showed that the FJT plans provided comparable target dose conformity, homogeneity, and coverage for the PTV50.4 and PTV60 to the SFT plans, although some parts of the PTV50.4 were covered by fewer beam directions in the FJT plans. This is consistent with the study of Chen et al.,^12^ which might be ascribed to two reasons. First, each part of PTV50.4 has been covered by a sufficient number of beam directions (at least six beams). Second, according to the attenuation and inverse‐square law, the part of the PTV50.4 further away from the radiation source will receive a relatively lower dose per MU. Therefore, the loss in dose coverage due to shielding these parts with the collimator jaws can be more efficiently compensated by other beam directions.^15^

In this study, the FJT plans also demonstrated a steeper dose fall‐off outside the PTV50.4 compared with the SFT plans. Our data showed that the volumes of the body‐PTV50.4 and lung receiving low and intermediate doses were significantly reduced with the FJT, and the other OARs, including the heart and spinal cord PRV, were also better spared compared to the SFT. This improved protection for OARs is in agreement with the findings from previous studies,^12^, ^14^, ^15^ but it is worth noting that our study achieved a higher degree of lung sparing than the study of Wang et al.^14^ In their study, the collimator jaws covered the entire target volume at each gantry angle for both the FJT and non‐FJT plan, and the reduction in the low‐dose volumes of the lung can only be ascribed to the leaf transmission reduction. In contrast, we shielded some parts of the PTV50.4 further away from the radiation source with the collimator jaws to further reduce the transmission in some beam directions in the FJT plan. Thus, the average path length across these fields was reduced concurrently. Biancia et al. has concluded that the integral dose for different beam arrangements is associated with the average path length.^21^ For these reasons, the steeper dose fall‐off outside the PTV50.4 in the FJT plans should be attributed to the combined effect of the reduction in the transmission and average path length. This dosimetric advantage, which can reduce the integral dose to the adjacent OARs and thus may lower the risk of normal tissue complications and secondary cancer,^22^ makes the FJT more favorable for treating cervical and upper thoracic EC patients. In addition, our results suggest that the metric AR can predict the dose gradient outside the target volume to some extent, but it might be influenced by other factors like the average path length of the treatment fields.

RP is the most common dose‐limiting complication in EC patients receiving external beam radiotherapy. Multiple DVH‐based parameters were identified to be associated with the incidence and severity of RP in the literature, such as the V_20Gy_, V_30Gy_, D_mean_.^10^, ^11^ Recent studies have found the volumes of the lung exposed to low doses might be more predictive of RP in EC patients receiving radiotherapy. Tanabe et al. performed a retrospective study of 86 EC patients undergoing definitive chemoradiotherapy, and found that the V_5Gy_ and V_10Gy_ values were the only factors significantly correlated with grade 2 or higher RP.^8^ Similarly, Shaikh et al. also reported that V_5Gy_ and V_10Gy_ were strong predictors of symptomatic RP in a cohort of 139 EC patients treated with chemoradiotherapy and concluded that minimizing the low dose spread to the lung can decrease the risk of RP for these patients.^9^ In this study, the FJT plans significantly reduced the volume of the lung treated at each dose level (5–40 Gy), especially below 13 Gy. These results indicate that the FJT may provide an advantage over SFT in lowering the risk of RP for cervical and upper thoracic EC patients receiving IMRT.

As an advanced form of IMRT, volumetric‐modulated arc therapy (VMAT) has been compared with static gantry IMRT (SG‐IMRT) regarding lung sparing for cervical and upper thoracic EC in the literature with a similar prescribed dose to this study. VMAT has demonstrated a reduction in the lung V_20Gy_ (0.3–3.8 points) and V_30Gy_ (0.7–2.4 points) at the cost of the increase in V_5Gy_ (0.5–4.7 points), V_10Gy_ (1.9–6.1 points), and V_13Gy_ (3.1 points) compared with SG‐IMRT.^23^, ^24^, ^25^ In combination with our data, it can be reasonably speculated that SG‐IMRT with FJT will further reduce the lung V_5Gy_, V_10Gy_, and V_13Gy_ in comparison to VMAT. Meanwhile, the difference in the lung V_20Gy_ and V_30Gy_ could be even smaller. Jaw tracking techniques (JTT), which continuously adjust the jaw positions to enclose the MLC apertures during the treatment delivery, is another approach to reducing MLC transmission for IMRT. Feng et al. reported that the lung V_5Gy_, V_10Gy_, V_20Gy_, and D_mean_ were reduced by an average of 2.6 points, 1.3 points, 0.6 points, and 0.4 Gy for thoracic cases with JTT compared to static jaw technique SG‐IMRT.^26^ However, it had no advantages over FJT in terms of lung sparing. For cervical and upper thoracic EC patients, SG‐IMRT with the FJT can serve as an alternative to VMAT and the JTT in terms of lung sparing, especially when these more advanced IMRT techniques are unavailable.

Compared to SFT, FJT required more manual operations before the plan optimization, and it usually took <10 min to manually split the PTV50.4 and adjust the jaw positions with the presented FJT procedure. Considering that the subsequent optimization process often consumed hours of human effort, the extension of treatment planning time in the FJT plans was relatively limited and acceptable. The treatment delivery efficiency is another major concern in IMRT. Decreasing the delivery time may not only improve patient throughput but also reduce the patients’ discomfort and the probability of patient movement during treatment.^15^, ^25^ Moreover, it has been found that the biological effect of radiotherapy decreased with elongation of delivery time.^27^ Similar to the results from the study by Lee et al.,^15^ our data showed that the FJT reduced the IMRT plan complexity by significantly lowering the number of MUs and subfields. Although the delivery time was only slightly shortened by 0.23 min because of the increased time of collimator rotation, the proposed FJT method still demonstrated a relative advantage over the SFT in the treatment delivery efficiency for cervical and upper thoracic EC patients with large target volumes.

The limitations of this study should be considered as well. FJT consistently reduced the low‐ and intermediate‐dose exposure of normal tissues while maintaining the target coverage and delivery efficiency compared with SFT for all of the 15 patients. However, for one patient with smaller PTV50.4 volume (237.32 cm^3^), the improvements in the OAR sparing using FJT (e.g., a reduction in the lung V_5Gy_ of 1.38 percentage points) were less evident compared with the other patients because the PTV50.4 of this patient extended less inferiorly to the upper thoracic region. This result indicates that these patients may benefit less from the presented method. Second, a quantitative metric that can measure the average path length across the entire field needs to be defined in the future in order to quantify the effect of the change in the average path length on reducing the low‐dose exposure. Finally, further studies are warranted to determine if the dosimetric benefits of the proposed technique will translate into improved clinical outcomes for cervical and upper thoracic EC.

## 5 CONCLUSIONS

The FJT combined with target splitting can provide superior OAR sparing and similar target coverage without compromising delivery efficiency compared with the SFT and should be a preferred IMRT planning method for cervical and upper thoracic EC patients.

## CONFLICT OF INTEREST

The authors declare that they have no competing interests.
